# Machine learning-based diagnostic model of lymphatics-associated genes for new therapeutic target analysis in intervertebral disc degeneration

**DOI:** 10.3389/fimmu.2024.1441028

**Published:** 2024-12-04

**Authors:** Maoqiang Lin, Shaolong Li, Yabin Wang, Guan Zheng, Fukang Hu, Qiang Zhang, Pengjie Song, Haiyu Zhou

**Affiliations:** ^1^ Department of Orthopedics, The Second Hospital & Clinical Medical School, Lanzhou University, Lanzhou, Gansu, China; ^2^ Key Laboratory of Bone and Joint Disease Research of Gansu Province, Lanzhou, Gansu, China

**Keywords:** intervertebral disc degeneration, machine learning, diagnostic model, lymphatic-associated gene, therapeutic target

## Abstract

**Background:**

Low back pain resulting from intervertebral disc degeneration (IVDD) represents a significant global social problem. There are notable differences in the distribution of lymphatic vessels (LV) in normal and pathological intervertebral discs. Nevertheless, the molecular mechanisms of lymphatics-associated genes (LAGs) in the development of IVDD remain unclear. An in-depth exploration of this area will help to reveal the biological and clinical significance of LAGs in IVDD and may lead to the search for new therapeutic targets for IVDD.

**Methods:**

Data sets were obtained from the Gene Expression Omnibus (GEO) database. Following quality control and normalization, the datasets (GSE153761, GSE147383, and GSE124272) were merged to form the training set, with GSE150408 serving as the validation set. LAGs from GeneCards, MSigDB, Gene Ontology, and KEGG database. The Venn diagram was employed to identify differentially expressed lymphatic-associated genes (DELAGs) that were differentially expressed in the normal and IVDD groups. Subsequently, four machine learning algorithms (SVM-RFE, Random Forest, XGB, and GLM) were used to select the method to construct the diagnostic model. The receiver operating characteristic (ROC) curve, nomogram, and Decision Curve Analysis (DCA) were used to evaluate the model effect. In addition, we constructed a potential drug regulatory network and competitive endogenous RNA (ceRNA) network for key LAGs.

**Results:**

A total of 15 differentially expressed LAGs were identified. By comparing four machine learning methods, the top five genes of importance in the XGB model (MET, HHIP, SPRY1, CSF1, TOX) were identified as lymphatics-associated gene diagnostic signatures. This signature was used to predict the diagnosis of IVDD with strong accuracy and an area under curve (AUC) value of 0.938. Furthermore, the diagnostic model was validated in an external dataset (GSE150408), with an AUC value of 0.772. The nomogram and DCA further prove that the diagnosis model has good performance and predictive value. Additionally, drug regulatory networks and ceRNA networks were constructed, revealing potential therapeutic drugs and post-transcriptional regulatory mechanisms.

**Conclusion:**

We developed and validated a lymphatics-associated genes diagnostic model by machine learning algorithms that effectively identify IVDD patients. These five key LAGs may be potential therapeutic targets for IVDD patients.

## 1 Introduction

The Global Burden of Disease study has reported that low back pain is one of the major causes of disability-adjusted life-years (DALYs) worldwide (1). Low back pain is a multifactorial outcome, and intervertebral disc degeneration (IVDD) is considered to be one of the major causes of low back pain. As evidenced by statistical analysis, discogenic low back pain constitutes approximately 26%–42% of cases (2). At present, the pathogenic mechanisms underlying intervertebral disc degeneration remain unclear. Identifying the various predisposing factors and developing diagnostic markers with etiologic specificity are the most pressing research priorities at this stage. These biomarkers can provide effective strategies for precise prevention and targeted treatment of IVDD.

The lymphatic system is part of the circulatory system of the body that is primarily responsible for the discharge of extracellular fluid containing cells, high- and low-molecular-weight proteins, and other molecules in the tissues (3). The normality of the lymphatic system determines the structure and reflux function of the lymphatic vessels (4, 5). Many diseases are associated with the function of the lymphatic system. For instance, the role of lymphatic reflux has been demonstrated in musculoskeletal disorders, such as osteoarthritis. Modulating lymphatic reflux has been shown to significantly reduce the progression of inflammatory arthritis (6, 7). Furthermore, an increasing number of studies have indicated that lymphatic vessels are closely linked to the development of IVDD. It has been demonstrated that with the progression of IVDD, lymphatic vessels comprising LYVE1+/podoplanin+ accompany the growth of vascularized fibrous tissue into the interior of the intervertebral disc (IVD) (8, 9). In addition, during IVDD, a variety of proteases (e.g., matrix metalloproteinases and aggrecan) and cytokines in the degenerating IVD stroma show elevated expression in response to the lack of lymphatic vessel supply, accelerating IVD prolapse (10, 11). At the same time, these factors also stimulate the proliferation and migration of blood and lymphatic vessels into the protruding IVD tissue in an autocrine manner (9). In addition, the lymphatic vasculature also plays an immunomodulatory role (12), and there is an apparent correlation between the progression of IVDD and immune cell infiltration (13). Thus, it is evident that lymphatic vessels play a crucial role in the IVDD process. However, the study of its underlying pathological mechanisms is still in its early stages. Further research into the biological significance of lymphatic-associated genes (LAGs) in IVDD may help accelerate the progress of molecular diagnosis and targeted therapy for this condition.

The selection of effective features is a crucial step in the discovery of biomarkers, which represents a fundamental task in this field of research. Machine learning is an effective strategy to address this problem, with its enormous resources to handle large, complex and diverse data, and has been applied to genomics research (14). Meanwhile, machine learning has enhanced our capacity to extract pertinent features from copious amounts of high-dimensional data pertaining to gene expression profiles (15), and has been extensively employed in medical biomarker screening (16, 17). However, there is still an unmet need for machine learning in the field of diagnosis and treatment of IVDD. In this study, based on the transcriptome data set and lymphatic vessel-related genes of IVDD patients obtained from the public database, we identified differentially expressed LAGs by bioinformatics analysis, and then used four machine learning algorithms to screen out lymphatic vessel gene biomarkers related to IVDD diagnosis, and used them as model genes to construct a diagnostic model. In addition, we also constructed the drug regulatory network and competitive endogenous RNA (ceRNA) network of model genes. The ultimate goal is to reveal the correlation between lymphatic vessel-related genes and the pathogenesis of IVDD and to provide a reference for potential drugs and candidate targets in IVDD treatment.

## 2 Materials and methods

### 2.1 Dataset download and data preprocessing

The original gene expression profile data is derived from the Gene Expression Omnibus (GEO) database (https://www.ncbi.nlm.nih.gov/geo/), which is an international public repository. We identified three training sets (GSE124272, GSE147383, and GSE153761) and one validation set (GSE150408). GSE124272 included eight patients with IVDD and eight normal controls, GSE147383 included four patients with IVDD and four normal controls, and GSE153761 included three patients with cervical spondylotic myelopathy and three healthy subjects. GSE150408 included seventeen patients with sciatica and seventeen healthy volunteers. This data was mainly used to confirm the analysis results.

The GSE124272, GSE147383, and GSE153761 datasets were integrated by the “limma” and “sva” packages in R (4.4.0), and the combined dataset was used as our training set. The “normalizbetween-arrays” function in the “limma” package of R language was used to normalize the expression matrix of the training set and the validation set, and the gene probes were annotated. A total of 302 LAGs were obtained from GeneCards (https://www.genecards.org/), MSigDB (https://www.gsea-msigdb.org/), Gene Ontology (https://geneontology.org/) and KEGG (https://www.kegg.jp/kegg/).

### 2.2 Characterization of immune infiltrating cells in the training set

We used twenty-two immune cells identified in previous studies (18, 19), analyzed the immune cell content of each sample in the training set using the “CIBERSORT” function in the “preprocessCore” package, and then The number of immune infiltrating cells, differential expression level, and correlation were visualized for each sample.

### 2.3 Identification and visualization of DEGs

DEGs within the training set were identified using the “limma” package, with screening criteria set at |log fold change (FC)| > 0.5 and P value < 0.05. Subsequently, visualizations of volcano and heat maps were created with the R package using ggplot2 and pheatmap, respectively.

### 2.4 Identification and functional enrichment of differentially expressed LAGs

The overlap of DEGs and LAGs within IVDD was obtained using the “VennDiagram” package in R, defining them as differentially expressed lymphatics-associated genes (DELAGs). Subsequently, the DELAGs underwent Gene Ontology (GO) analysis, Kyoto Encyclopedia of Genes and Genomes (KEGG) analysis, Disease Ontology (DO) analysis, and Gene Set Enrichment Analysis (GSEA) using the “clusterProfiler” package in R. Enrichment results with a P-value less than 0.05 were considered statistically significant.

### 2.5 Screening of model genes by machine learning algorithms

Fifteen LAGs exhibiting differential expression were analyzed via machine learning algorithms. Four machine learning algorithms, including Extreme Gradient Boosting (XGB), Random Forest (RF), Support Vector Machine (SVM), and the Generalized Linear Model (GLM), were employed for the selection of key genes in IVDD diagnosis. We trained RF, SVM, XGB, and GLM models using the train function from the R “caret” package, in conjunction with the “randomForest”, “kernlab”, “xgboost”, and “stats” packages, respectively. Meanwhile, we leveraged the built-in grid search mechanism of the “caret” package to explore the optimal hyperparameter combinations (**Table 1**) and evaluated the performance of each combination through cross-validation. Additionally, residual boxplots, feature importance plots, reverse cumulative distribution of residuals, and Receiver Operating Characteristic (ROC) curves were constructed for XGB, RF, SVM, and GLM models to ascertain model genes.

**Table 1:** Hyperparameter tuning settings for each machine learning algorithm.

Machine learning algorithm	Hyperparameters
RF	mtry: 2, ntree: 500
SVM	sigma: 0.04, c: 0.25
XGB	nrounds: 100, max_depth: 2, eta: 0.4, gamma: 0, colsample_bytree: 0.8, min_child_weight: 1, subsample: 0.5
GLM	no specific

### 2.6 Model construction and validation

Diagnostic model nomograms were constructed using model genes and the “rms” package in R software, with calibration curves employed to validate their accuracy. The efficacy of the Nomo-clinical grading was assessed through Decision Curve Analysis (DCA). ROC curves were plotted to evaluate the diagnostic value of model genes in IVDD. Subsequently, an external validation cohort (GSE150408) was introduced to assess the diagnostic capability and robustness of the model.

### 2.7 GSVA enrichment analysis of model genes and its correlation with immune cells

To unravel the potential biological functions of the model genes, we performed Gene set variation analysis (GSVA) by using the “clusterprofiler” package in the GO and KEGG genomes. Reference gene sets included c2.cp.kegg.Symbols.gmt and c5.go.Symbols.gmt. Enrichment results with a P-value less than 0.05 were deemed statistically significant. The “GSVA” package in R was employed to investigate the association between model genes and twenty-eight immune cells, with visualization of the results carried out using the “ggplot2” package.

### 2.8 Construction of drug regulatory networks and ceRNA networks

Interactions between model genes and drugs were obtained using the Drug-Gene Interaction database (DGIdb) (https://www.dgidb.org/), and the results were visualized using Cytoscape software. The results were analyzed using miRanda (http://www.microrna.org/), miRDB (http://mirdb.org/), miRWalk (http://mirwalk.umm.uni-heidelberg.de/), and TargetScan (http://www.targetscan.org/) databases and predicted microRNAs (miRNAs) targeting model genes using Score=1 as a screening condition. SpongeScan database (http://spongescan.rc.ufl.edu/) was employed for predicting lncRNA-miRNA interactions (20). Subsequently, Cytoscape software was utilized for the creation and visualization of lncRNA-miRNA-mRNA regulatory networks.

### 2.9 Statistical analysis

All data processing, plotting, and statistical analysis are performed in R software (4.4.0). Differences between independent variables and non-normally distributed variables were analyzed using the Wilcoxon test. The correlation between the two continuous variables was determined by Spearman correlation analysis. ROC curve analysis was performed using the “pROC” package in R software. P < 0.05 was considered statistically significant.

## 3 Results

### 3.1 The landscape of immune infiltration in IVDD

**Figure 1:**
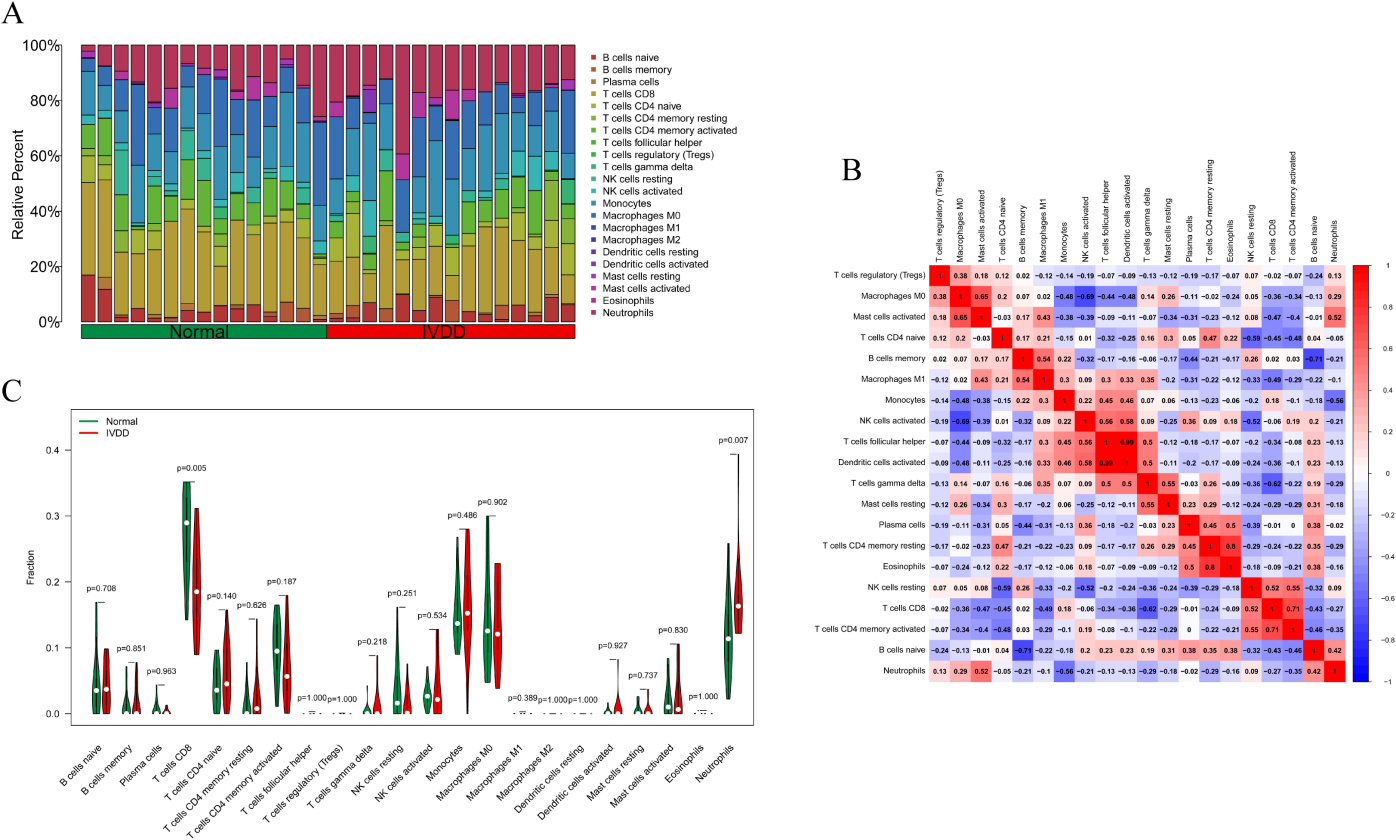
Examination of immune cell infiltration in the normal group and IVDD group in the training set. **(A)** A bar chart of the proportion of 22 immune cells in the normal group and the IVDD group; **(B)** The relationship between immune infiltrating cells in the training set. Red indicates a positive relationship, blue indicates a negative relationship, with darker colors representing a stronger relationship; **(C)** A violin diagram of the difference in the content of 22 immune cells between the normal group and the IVDD group. Statistically significant when p < 0.05. IVDD, intervertebral disc degeneration.

Based on the merged training set from the GSE124272, GSE147383, and GSE153761 datasets, the relative proportions of 22 immune cell subsets in the normal control group and the IVDD group were assessed using CIBERSORT technology (**Figure 1A**). Subsequently, further analysis was conducted to decode the interrelationships among these infiltrating immune cells (**Figure 1B**). The results revealed strong positive correlations between T cells follicular helper and dendritic cells activated (r = 0.99), T cells CD4 memory resting and eosinophils (r = 0.8), and T cells CD8 and T cells CD4 memory activated (r = 0.71). On the contrary, there were notable negative correlations between activated NK cells and M0 macrophages (r = -0.69), T cells gamma delta and T cells CD8 (r = -0.62), and B cells naïve and B cells memory (r = -0.71). As depicted in the violin plot (**Figure 1C**), CD8 T cell infiltration was significantly lower (*p* = 0.005) and neutrophil infiltration was significantly higher (*p* = 0.007) in the IVDD group compared to the normal control group, indicating that immune cells are involved in the progression of IVDD.

### 3.2 Identification of DEGs in the IVDD and normal groups

**Figure 2:**
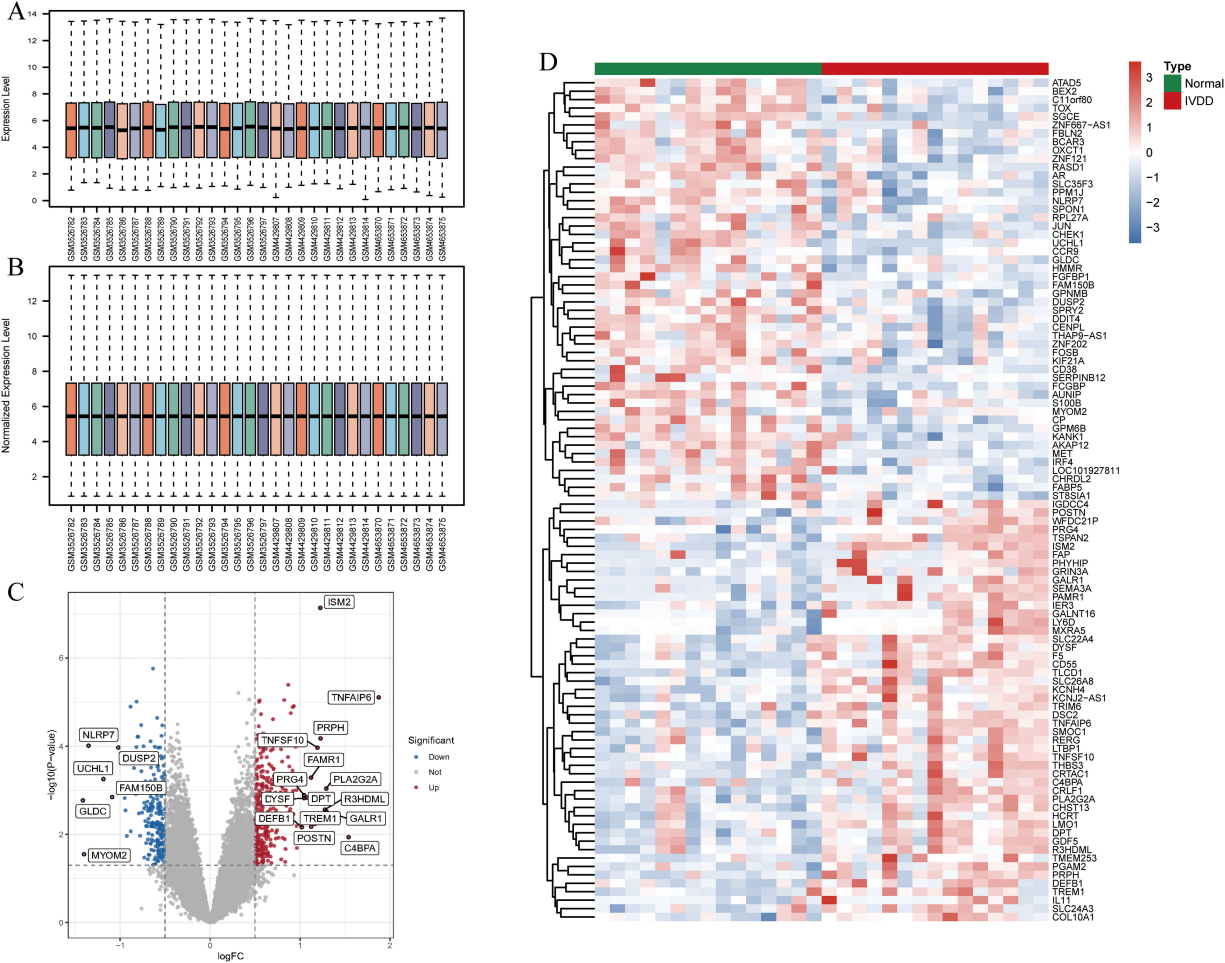
Identification and analysis of DEGs in the training set. **(A)** The bar chart of the expression matrix of 30 samples in the training set before normalization; **(B)** The bar chart of the expression matrix in 30 samples in the training set after normalization; **(C)** Volcano plot of DEGs expression. Red indicates genes with increased expression, grey is non-significant, and green indicates genes with decreased expression; **(D)** Heatmap of DEGs expression. Red is the high expression and green is the low expression. DEGs, differentially expressed genes; IVDD, intervertebral disc degeneration.

GSE124272 comprises 16 samples, GSE147383 includes 8 samples, and GSE153761 contains 6 samples. After merging these three datasets, the training set consists of 30 samples. Subsequently, the expression matrix data of the 30 samples were normalized (**Figures 2A, B**). A total of 517 differentially expressed genes (DEGs), including 306 upregulated genes and 211 downregulated genes, were identified between the normal and IVDD groups. The volcano plot depicting the expression of DEGs is shown in **Figure 2C**, with red representing higher expression levels, green indicating lower expression levels, and all other genes depicted in gray. **Figure 2D** displays the heatmap of the top 50 genes with increased (red) and decreased (green) expression levels based on logFC between the normal and IVDD groups.

### 3.3 Identification and functional analysis of DELAGs

**Figure 3:**
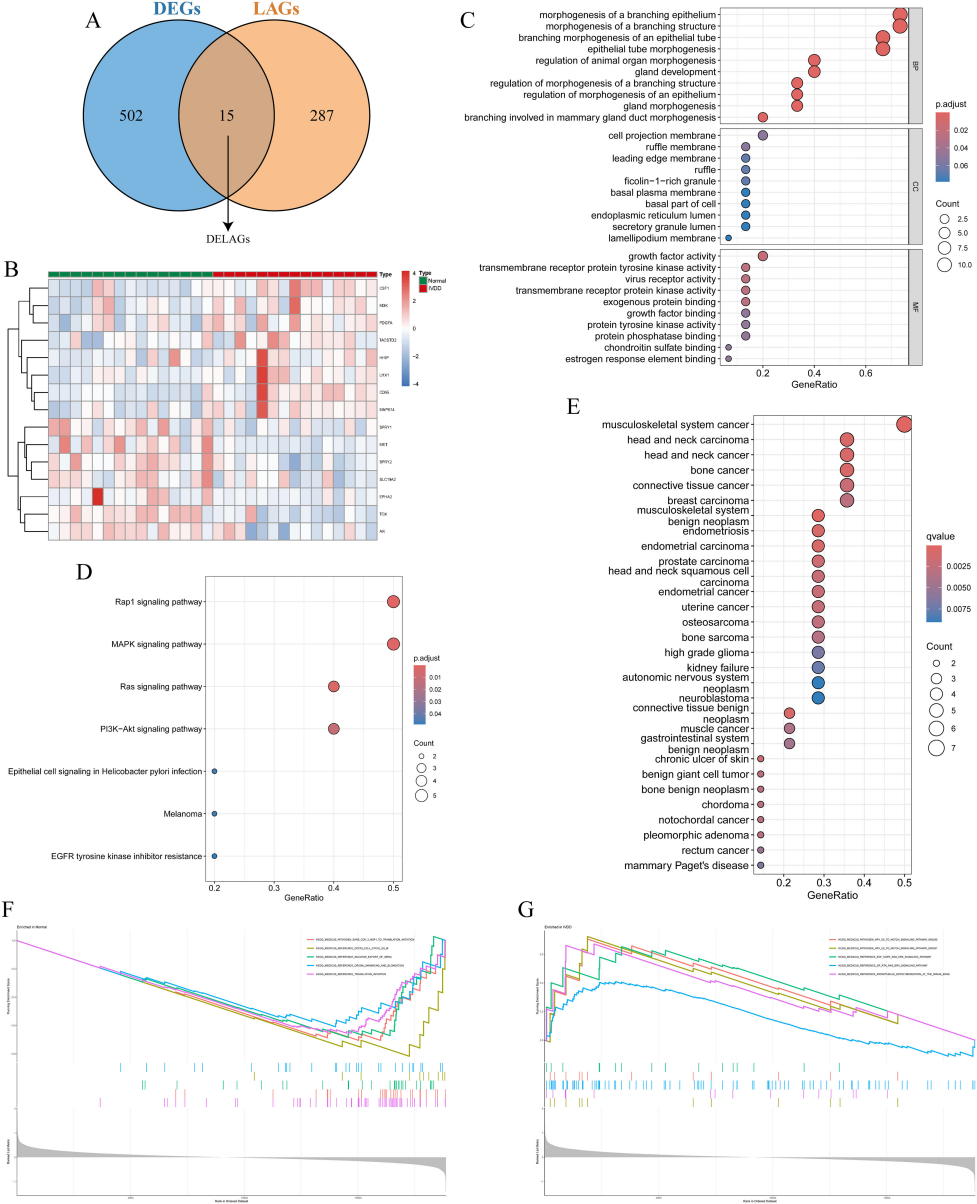
Identification and functional analysis of DELAGs. **(A)** Veen diagram shows the intersection of DEGs and LAGs; **(B)** Heat map of DELAGs differential expression in normal group and IVDD group; **(C)** The bubble diagram of GO enrichment analysis of DELAGs, including BP, CC and MF; **(D)** Bubble diagram of KEGG enrichment analysis of DELAGs; **(E)** Description of DO enrichment analysis results of DELAGs; **(F)** GSEA analysis results of DELAGs in the normal group; **(G)** GSEA analysis results of DELAGs in the IVDD group. DEGs, differentially expressed genes; LAGs, lymphatics-associated genes; DELAGs, differentially expressed lymphatics-associated genes; GO, gene ontology; BP, biological process; CC, cellular component; MF, molecular function; KEGG, Kyoto Encyclopedia of Genes and Genomes; DO, disease ontology; GSEA, gene set enrichment analysis.

Using keywords such as “lymphatic” and “lymphatic vessel,” we retrieved 302 LAGs from GeneCards, MSigDB, Gene Ontology, and KEGG databases. Intersection with the DEGs in the training set identified 15 DELAGs (**Figures 3A, B**). To elucidate the biological functions of these DELAGs, we employed GO, KEGG, and DO analysis tools. GO enrichment analysis revealed that DELAGs are mainly enriched in processes such as morphogenesis and regulation of branching epithelium and structure, morphogenesis and development of glands, growth factor activity, transmembrane receptor protein tyrosine kinase activity, and exogenous protein binding (**Figure 3C**; **Supplementary Table S1**). The DELAGs also exhibited consistent trends with the Rap1, MAPK, Ras, and PI3K-Akt signaling pathways (**Figure 3D**; **Supplementary Table S2**). Further Disease Ontology analysis explored the associations of DELAGs with various diseases, including tumors of the musculoskeletal system and head and neck, as well as chronic ulcers of the skin (**Figure 3E**; **Supplementary Table S3**). GSEA analysis results revealed the enrichment of pathways such as Cell cycle and translation initiation in samples from the normal control group (**Figure 3F**), while the Notch and Ras/ERK signaling pathways were enriched in IVDD samples (**Figure 3G**).

### 3.4 Based on multiple machine learning algorithms to screen key DELAGs as model genes

**Figure 4:**
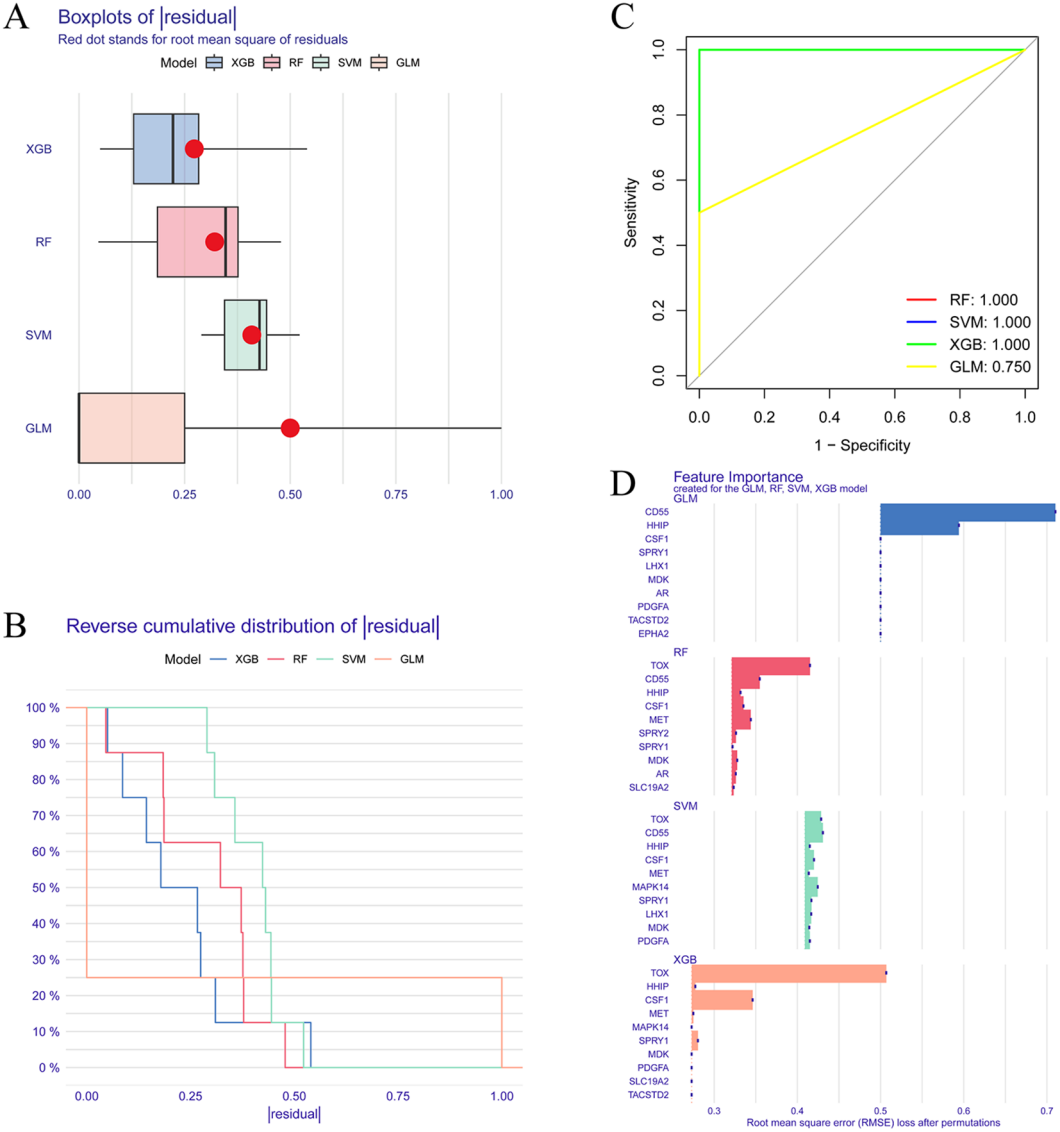
Screening of key DELAGs. **(A)** The box plots of residuals for the XGB, RF, SVM, and GLM models; **(B)** Reverse cumulative distribution of residuals in XGB, RF, SVM, and GLM models; **(C)** The ROC curve evaluates the diagnostic effect of XGB, RF, SVM and GLM models; **(D)** Feature Importance created for the GLM, RF, SVM, XGB model. XGB, Extreme Gradient Boosting; RF, Random Forest; SVM, Support vector machines; GLM, Generalized linear model.

We compare the four machine learning algorithms (XGB, RF, SVM, and GLM) by calculating the residual values (**Figure 4A**), the reverse residual distribution plots (**Figure 4B**), and the ROC curves (**Figure 4C**). The residual values are the smallest for XGB and the largest for GLM. The Area under Curve (AUC) values for XGB, RF, SVM, and GLM are 1.00, 1.00, 1.00, and 0.75. At the same time, we also calculated the accuracy, standard deviation of accuracy, Kappa value, and standard deviation of Kappa value of the four models. XGB was 0.853, 0.219, 0.704, and 0.444; SVM was 0.863, 0.237, 0716, 0.488; RF was 0.807, 0.298, 0.608, 0.6; GLM was 0.57, 0.388, 0.134, 0.768. By performing Friedman’s test and Nemenyi’s *post hoc* test on the accuracy and kappa values, we found that there is no significant difference in the performance of the three models, XGB, SVM, and RF (p > 0.05), whereas there is a significant difference between the GLM model and the three, and in particular, there is a significant difference between the XGB and the SVM with it (p < 0.01). Although XGB, SVM, and RF are not statistically different, when combined with specific values, the SVM model has the best accuracy and kappa value, while the XGB model is the most stable. In addition, we plotted the feature importance of the four models (**Figure 4D**). Based on the results of the above-combined comparison of the four models, we identified the XGB model as the best model. Then, we identified the top five genes in the XGB model as the key DELAGs (MET, HHIP, SPRY1, CSF1, and TOX) and used them as the model genes to construct a diagnostic model for predicting IVDD.

### 3.5 Model gene-based diagnostic model construction and diagnostic performance evaluation

**Figure 5:**
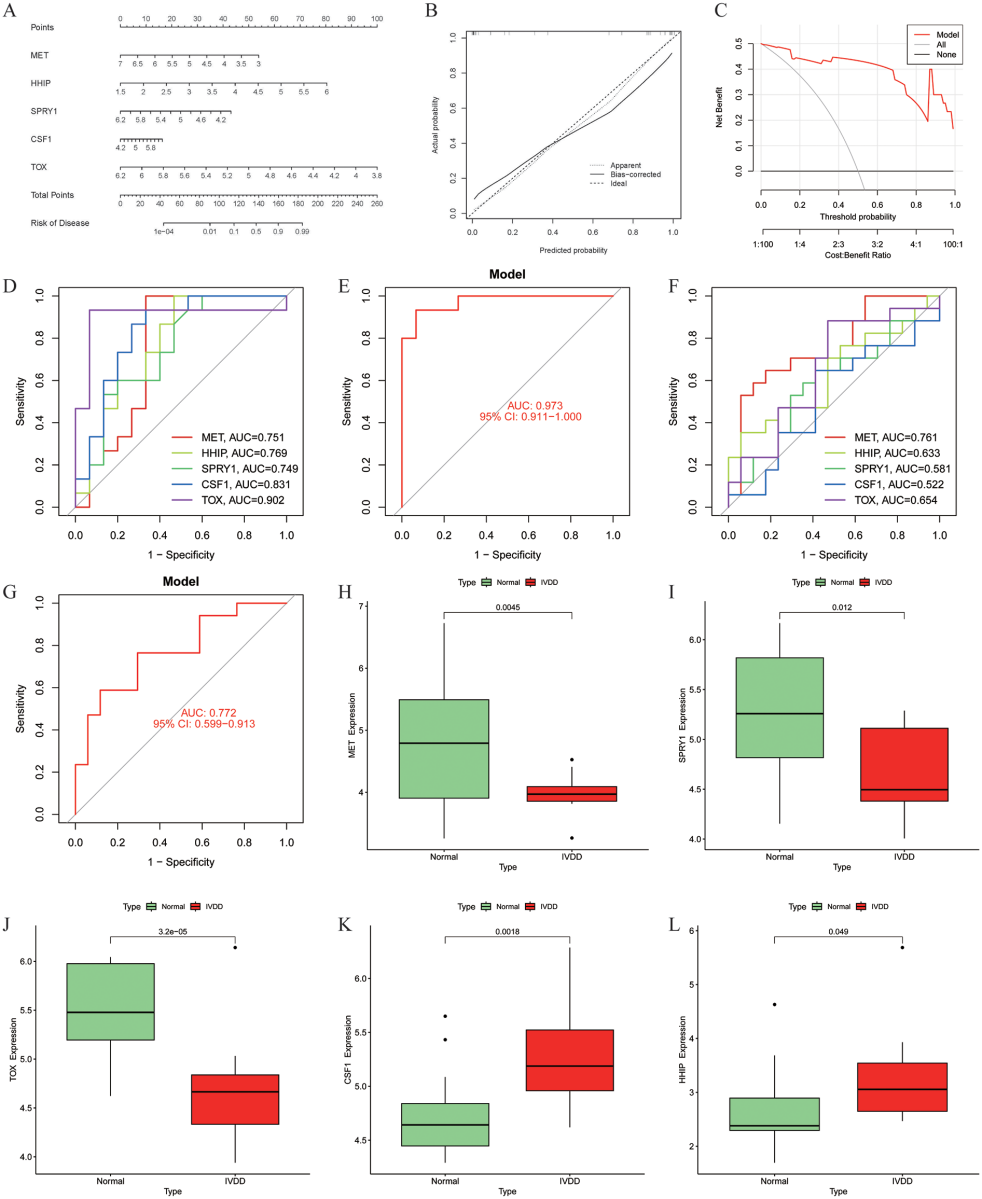
Construction and diagnostic value of the diagnostic model. **(A)** Model gene nomogram for the diagnosis of IVDD; **(B)** Calibration curve evaluation of the nomogram model; **(C)** DCA curves of the nomogram prediction; **(D)** ROC curves evaluating the diagnostic effect of five model genes in the training set; **(E)** The entire ROC curve for the five model genes in the training set; **(F)** ROC curves evaluating the diagnostic effect of five model genes in the validation set; **(G)** The entire ROC curve for the five model genes in the validation set; **(H–L)** Differential expression of model genes in the training set, MET (*p* = 0.0045), SPRY1 (*p* = 0.012) and TOX (*p* = 3.2 × 10-5) were lowly expressed in the IVDD group, and CSF1 (*p* = 0.0018) and HHIP (*p* = 0.049) were highly expressed in the IVDD group, *p* < 0.05 was statistically significant. DCA, Decision Curve Analysis; ROC, Receiver operating characteristic.

A nomogram model was built based on five model genes to investigate the risk of IVDD (**Figure 5A**). Evaluation of the nomogram model using calibration curves showed good consistency between the five model genes and the ideal model (**Figure 5B**), indicating the nomogram’s strong predictive value. The DCA results showed that the decision curve of the model was always higher than the None and ALL reference lines, indicating that the decision-making based on the nomogram may benefit IVDD patients, and the nomogram model has strong clinical utility (**Figure 5C**). Finally, separate ROC curves and an overall ROC curve were constructed using the five model genes in both the training and validation sets (**Figures 5D–G**). The results of the ROC curve analysis demonstrated that the constructed diagnostic model exhibited excellent predictive performance in the training set, as evidenced by an area under the curve (AUC) value of 0.973 (**Figure 5E**). Furthermore, the sensitivity, specificity, and accuracy of the model, as well as the positive predictive value (PPV) and negative predictive value (NPV), were all 0.933. Meanwhile, the Positive Likelihood Ratio (PLR) was as high as 14, while the Negative Likelihood Ratio (NLR) was 0.07. In the validation set, the AUC value of the diagnostic model was 0.772 (**Figure 5G**), and its sensitivity was 0.765, specificity 0.706, and accuracy 0.735. In addition, the PPV was 0.722, NPV 0.75, PLR 2.6, and NLR 0.33. Combining the above diagnostic performance metrics, the model demonstrated good accuracy and stability in diagnosing IVDD. Additionally, we visually represented the expression of the five model genes in the training set using boxplots (**Figures 5H–L**). The results revealed that MET, SPRY1, and TOX exhibited lower expression in the IVDD group compared to the normal control group, while CSF1 and HHIP showed higher expression in the IVDD group.

### 3.6 GSVA analysis of model genes and its correlation with immune cells

**Figure 6:**
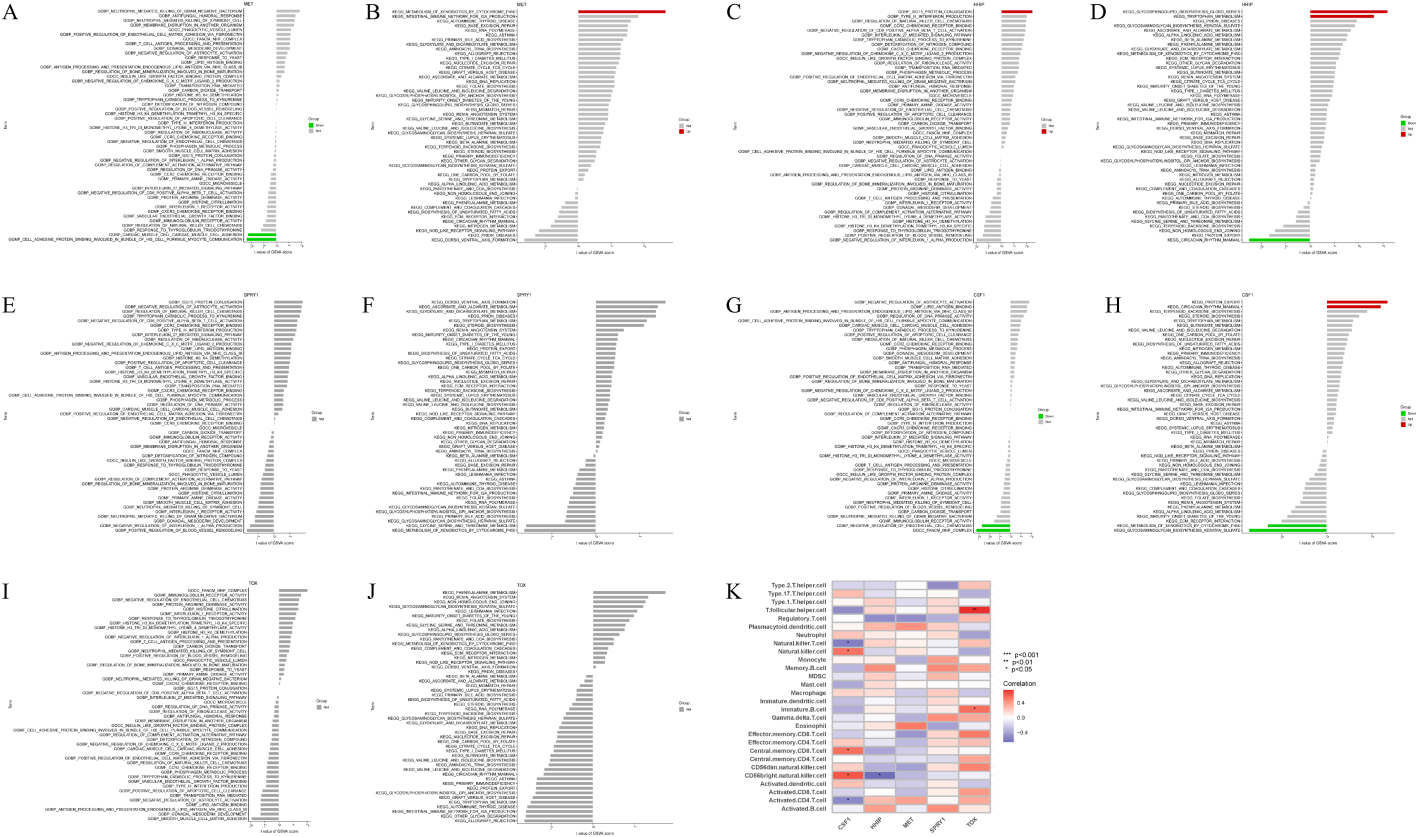
GSVA of model genes in the training set and their correlation with 28 immune cells. **(A, B)** GO and KEGG analysis in GSVA of MET; **(C, D)** GO and KEGG analysis in GSVA of HHIP; **(E, F)** GO and KEGG analysis in GSVA of SPRY1; **(G, H)** GO and KEGG analysis in GSVA of CSF1; **(I, J)** GO and KEGG analysis in GSVA of TOX; **(K)** Correlation between five model genes and 28 immune cells, red represents positive correlation, blue represents negative correlation, the darker the color the stronger the correlation. *p<0.05, **p<0.01, ***p<0.001.

In the training set, GSVA of the five model genes (**Figures 6A–J**) revealed the following: GO function in the MET low expression group was mainly enriched in the binding of cell adhesion protein involved in communication between the bundle of HIS cells and Purkinje myocytes (**Figure 6A**), and the high expression group of the KEGG pathway was mainly enriched in metabolism of xenobiotics by cytochrome p450 pathway (**Figure 6B**). The GO function of the HHIP high expression group was mainly enriched in ISG15 protein conjugation (**Figure 6C**), and the KEGG pathway was mainly enriched in glycosphingolipid biosynthesis globo series and tryptophan metabolism (**Figure 6D**). The GO function in the low CSF1 expression group was mainly enriched in the negative regulation of endothelial cell chemotaxis (**Figure 6G**), and the KEGG pathway was mainly enriched in metabolism of xenobiotics by cytochrome p450 and Glycosaminoglycan biosynthesis keratan sulfate, whereas the KEGG pathway in the high-expression group was mainly enriched in Protein export and circadian rhythms in mammals (**Figure 6H**). Moreover, the correlation between the five model genes and 28 immune cells was analyzed (**Figure 6K**). The analysis revealed that CSF1 was positively correlated with natural killer cells, central memory CD8 T cells, and CD56 bright natural killer cells while showing negative correlations with natural killer T cells and activated CD4 T cells. HHIP exhibited a significant negative correlation with CD56 bright natural killer cells. TOX had the strongest positive correlation with T follicular helper cells, followed by immature B cells. These findings suggest that these model genes are involved in the immune regulation processes during IVDD progression.

### 3.7 Construction of drug regulatory network and ceRNA network

**Figure 7:**
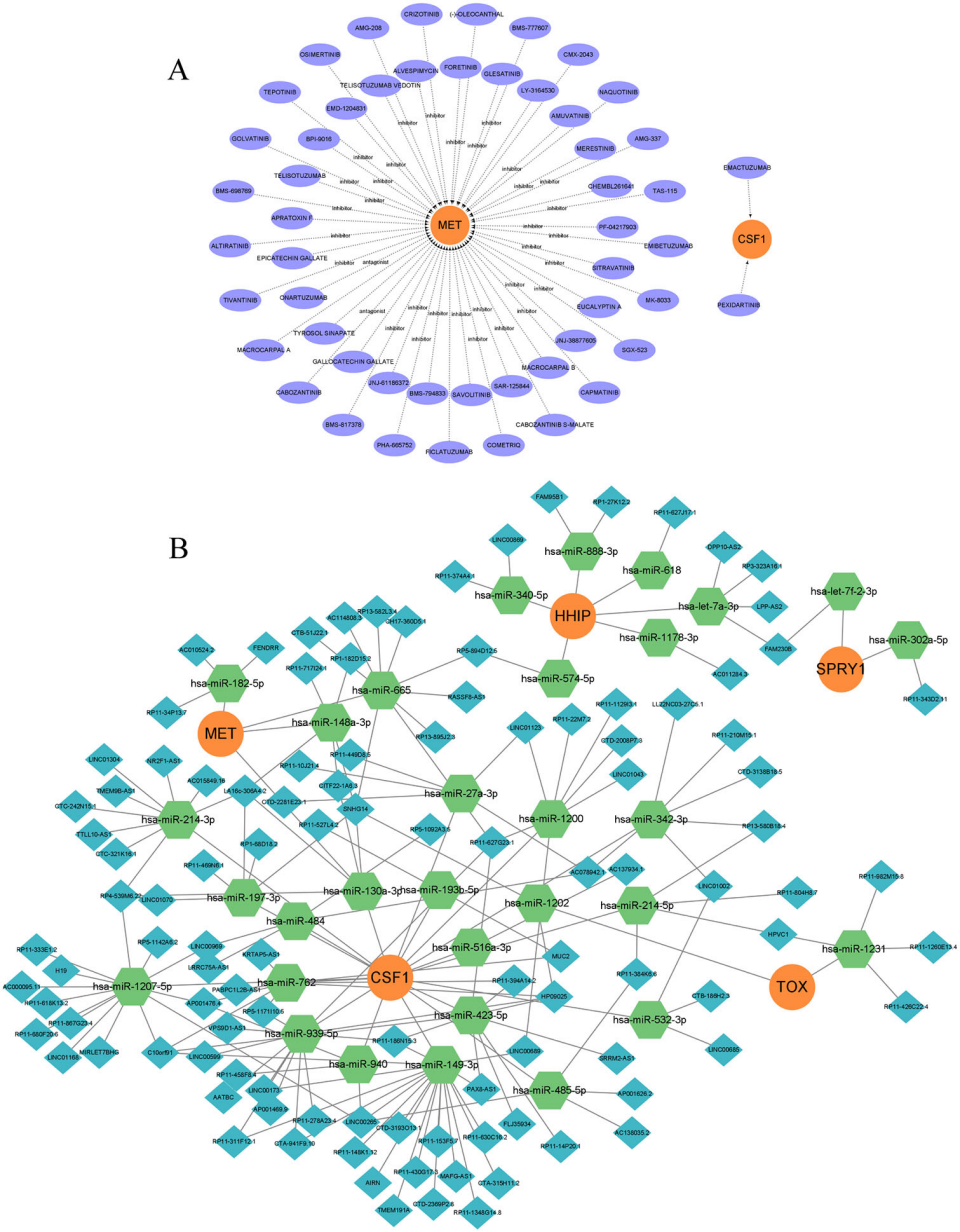
Drug regulatory network and ceRNA network. **(A)** Prediction of drug-gene interactions for model genes, orange represents model genes, purple represents predicted drugs; **(B)** ceRNA network, orange circle represents model gene, green hexagon represents miRNA, and blue diamond represents lncRNA.

Based on the five model genes, we identified potential therapeutic drugs using the gene-drug interaction data in DGIdb and demonstrated the interactions between the drugs and model genes using Cytoscape (**Figure 7A**). Information on the source, interaction type, and score of the target drugs is shown in **Supplementary Table S4**. According to the ‘ Interaction Score ‘ in DGIdb, a total of 52 potential drugs were identified, 50 drugs were targeted at MET, and 2 drugs were targeted at CSF1. No potential drugs for HHIP, SPRY1, and TOX were found. Additionally, to uncover the potential post-transcriptional regulatory mechanisms of the five model genes, a lncRNA-miRNA-mRNA regulatory network was constructed. miRNAs targeting the five model genes were predicted using miRanda, miRDB, miRWalk, and TargetScan, selecting those with a score of 1 across all four databases. SpongeScan was then used to predict lncRNA-miRNA interactions, and the complete ceRNA network was constructed using Cytoscape (**Figure 7B**). Within each lncRNA-miRNA-mRNA regulatory axis, lncRNAs can enhance the expression levels of the model genes by inhibiting the corresponding miRNAs. For instance, LINC00173 may indirectly upregulate CSF1 expression by suppressing miR-939-5p, thereby enhancing its role in regulating the survival, proliferation, and differentiation of hematopoietic progenitor cells. Overall, this ceRNA network helps to elucidate the potential post-transcriptional regulatory mechanisms in IVDD and provides a reference for subsequent therapeutic strategies.

## 4 Discussion

IVDD is a primary cause of lower back pain, with its etiology rooted in various factors such as genetic predisposition (21), mechanical stress (22), trauma (23), smoking (24), obesity (25), and aging (26). The extrusion of nucleus pulposus tissue during IVDD, which has autoantigenic properties, can trigger an immune-inflammatory response (27, 28), compressing the nerves or spinal cord, leading to immune cell infiltration of the intervertebral tissues (29–31), and causing low back pain that affects the mobility of both lower limbs (32). The lymphatic system, a crucial component of the circulatory system, is essential for maintaining tissue fluid balance, lipid absorption, immune surveillance, and the transport of immune cells (33). Current research generally agrees that the process of IVDD involves changes in the distribution of lymphatic vessels. Specifically, during the degeneration of a normal IVD, lymphatic vessels are accompanied by blood vessels from the surrounding tissues into the degenerated IVD tissue. The proteases, inflammatory cytokines, and growth factors present in the degenerated IVD matrix facilitate this process (9). Abnormal lymphatic vessel formation induced by IVDD may resemble that observed in lymphedema, obesity, and cancer, closely correlating with the occurrence and progression of the disease (34). Modulating lymphatic vessel formation could potentially become a future therapeutic target for IVDD. However, the mechanistic association between lymphatic vessels and IVDD remains unconfirmed, with a scarcity of relevant research. In this study, a diagnostic signature related to lymphatic vessels was generated by integrating gene expression profiles and LAGs using machine learning algorithms. The performance of the diagnostic model was evaluated using both training and external validation sets. In addition, GSVA was used to explore the potential mechanisms of the model genes and their correlation with immune cell infiltration and to predict potential drugs and ceRNA networks targeting the model genes. Our results may offer potential applications for IVDD patients, such as early diagnosis and the selection of new therapeutic targets.

In this study, three IVDD-related datasets were initially acquired from the GEO database and merged to form the training set, with GSE150408 serving as the validation set. Bioinformatic techniques were then used to examine the immune cell characteristics within the training set, revealing significant statistical discrepancies in CD8+ T cells and neutrophils. IVDD samples showed a significant increase in neutrophils and a marked decrease in CD8+ T cells compared to the control group. This is consistent with the findings of Kaneyama et al. (35) that nucleus pulposus (NP) tissue extruded from degenerated IVD induces an immune response, leading to infiltration of immune cells that disrupt the physiological barrier of IVD cells, resulting in NP cell apoptosis. We then screened DEGs and LAGs from the training set and four databases (GeneCards, MSigDB, Gene Ontology, KEGG), and obtained 15 DELAGs in the training set after taking the intersection.

Subsequently, functional enrichment analysis was performed on DELAGs. According to GO analysis, DELAGs were enriched in both BP and MF. BP mainly includes branching epithelial cell and structure morphogenesis, regulation of animal organ morphogenesis, branching structure, and epithelial cell morphogenesis. MF mainly includes growth factor activity, transmembrane receptor protein tyrosine kinase activity, virus receptor activity, transmembrane receptor protein kinase activity, exogenous protein binding, growth factor binding, protein tyrosine kinase activity, and protein phosphatase binding. KEGG enrichment analysis revealed that DELAGs primarily influenced the Rap1 signaling pathway, MAPK signaling pathway, Ras signaling pathway, and PI3K-Akt signaling pathway in the IVDD group. The Ras signaling pathway is closely linked to inflammatory responses (36). Both the MAPK signaling pathway (23) and PI3K-Akt signaling pathway have been extensively linked to the occurrence and progression of IVDD. PI3K-Akt signaling pathway is involved in IVD cell proliferation, senescence, and apoptosis, and activation of this pathway can delay the progression of IVDD by upregulating SOX9 expression (37). Furthermore, studies indicate that cyclic mechanical stretching can ameliorate NP cell degeneration via the PI3K-Akt signaling pathway (38–40). GSEA unveiled that the genes within the IVDD group are predominantly associated with the Notch and Ras/ERK signaling pathways. Notably, a study delineated that the activation effects of Notch signaling exert cell type-specific influences on genes regulating IVD matrix synthesis and degradation metabolism (41). Specifically, the activation of Notch signaling can promote the expression of matrix degradation genes in anulus fibrosus (AF) and ATDC5 cells and inhibit the expression of matrix anabolism genes. In NP cells, this effect inhibited the expression of matrix degradation genes (including MMP3, MMP13, Adamts4, and Adamts5), and attenuated the expression of MMP13 induced by TNF-α and macrophages. In addition, it has been demonstrated that the expression of Notch signaling molecules and their downstream target genes can be detected in AF and NP cells in IVD tissues (42), with a significant increase in Notch signaling activity noted in degenerated IVD tissue compared to healthy human IVD (43).

In comparison to conventional statistical models, machine learning can provide better predictive performance and capture the complex interactions among predictors and the non-linear relationship between predictors and outcomes (44). Based on this, we employed four machine-learning algorithms and identified the top 5 genes (MET, HHIP, SPRY1, CSF1, and TOX) in the XGB model as model genes through comparative analysis. To validate the reliability of these model genes, we constructed a diagnostic model for predicting IVDD utilizing these five model genes. It is necessary to note that evaluation of model performance and external validation are imperative for the constructed model (45). The dataset utilized for external validation should also differ from the training set in terms of both temporality and geography, as external validation constitutes the model’s redevelopment process on a validation set (46, 47). Calibration and discrimination are some of the most basic and important indicators for model performance (48). Calibration refers to the degree of consistency between the predicted risk of predictors and the observed results, which can be evaluated by calibration curves (49). Within this study, the calibration curve of the diagnostic model closely matches the observed results curve, indicating that the diagnostic model we constructed has a high predictive ability for IVDD. Concurrently, as a calibration supplement, we introduced the DCA, a method to determine whether the information provided by the prediction model for decision-making in clinical practice is more beneficial than harmful (50). In this study, the DCA of the model gene in the training set consistently favors patients, demonstrating the model gene’s stable diagnostic value for IVDD. Moreover, the model must also possess the discriminatory ability to differentiate between true positives and true negatives, typically assessed using the C-index, which is akin to the AUC in ROC; higher values indicate a stronger ability of the model to distinguish between true positives and true negatives (51). The C-indexes of the model genes in the training and validation sets were 0.973 and 0.772, respectively, and the accuracy was 0.933 and 0.735, respectively. These findings suggest that the diagnostic model constructed based on the model genes has a high degree of confidence in distinguishing between the normal group and the IVDD group. Notably, the overall efficacy of the model gene surpasses that of the five individual model genes, which is consistent with the multi-molecular driving properties of IVDD. Moreover, the expression levels of these five model genes in the original data display significant disparities between the normal and IVDD groups, with MET, SPRY1, and TOX demonstrating lower expression in IVDD samples compared to the normal group, potentially acting as protective genes for IVDD, while CSF1 and HHIP exhibit increased expression in IVDD samples, which could be used as the risk gene of IVDD. Thymocyte Selection Associated High Mobility Group Box (TOX) is a nuclear DNA-binding protein that plays an important role in the development of CD4+ T cells, NK cells, and intrinsic lymphocytes and is a key regulator of Exhausted T cell development (52). T-cell exhaustion is a pervasive phenomenon that serves to prevent immune overactivation and establish immune homeostasis in response to chronic inflammatory stimuli while limiting T-cell-mediated immunopathology (53). In this process, TOX induces CD4+ T cells to produce IL-10, thereby regulating inflammation (54). The exogenous addition of IL-10 has been demonstrated to alleviate IL-1β-induced degeneration of NP cells. Furthermore, IL-10 treatment has been shown to significantly suppress mRNA expression of type X collagen, as well as degradation of aggrecan and type II collagen, through the inhibition of the p38/MAPK pathway. In addition, IL-10 up-regulates mRNA expression of SOX9, exerting a protective effect against IVD (55). Hedgehog interacting protein (HHIP) is a type I transmembrane glycoprotein that plays a role in a number of biological processes, including development and angiogenesis (56, 57). Cross-sectional studies have found that circulating HHIP levels are significantly elevated in overweight/obese women and positively correlate with blood glucose, insulin, and body mass index, while HHIP levels are regulated by blood glucose and insulin levels. Obesity is currently acknowledged as an independent risk factor for the onset of Intervertebral Disc Disease (IVDD) and is significantly correlated with elevated levels of IL-6 and systemic pro-inflammatory cascades. Therefore, increased circulating levels of HHIP may be linked to the activation of inflammatory pathways associated with IVDD degeneration. Intervertebral disc degeneration (IVDD) is a multifactorial disease. Genetic susceptibility, mechanical stress, obesity, and smoking have all been identified as risk factors for IVDD. While there is limited research directly examining the association between the aforementioned model genes and IVDD, further investigation into the pathogenesis of IVDD and a more detailed analysis of the functions of the five model genes may gradually elucidate the interactions between them.

To further explore the biological functions of the model gene, we conducted Gene Set Variation Analysis (GSVA) and immune cell correlation analysis. The results revealed that CSF1 is primarily enriched in the biosynthesis process of glycosaminoglycans (GAG). It has been established that GAG plays a pivotal role in the human IVD. During IVDD, proteoglycans containing GAG are degraded and depleted, resulting in the loss of extracellular matrix integrity (58), and abnormal changes in GAG chain replacement during degeneration exacerbate this process (59). Hence, to maintain normal IVD function, high concentrations, and highly charged proteoglycans must be sustained, enabling functional proteoglycans to possess as many sulfated GAG chains as possible. However, enzymes involved in GAG biosynthesis (such as XT-1 and GlcAT-I) are downregulated with age and degeneration, resulting in weakened proteoglycan function and abnormal elongation of GAG chains (60, 61). Thus, we hypothesize that CSF1 mediates pathological changes in IVD by regulating GAG biosynthesis. Research has shown that during IVDD, immune cells, and their inflammatory factors can infiltrate the IVD through defects in the cartilaginous endplate and AF, accelerating catabolism and inducing inflammatory responses (62). Therefore, immunotherapy targeting immune cells is viewed as a novel strategy to alleviate IVDD (63). Meanwhile, genes associated with immune cells may also serve as biomarkers for IVDD (64). Based on this, we analyzed the inter-association between model genes and 28 immune cells. The findings revealed significant correlations between TOX, CSF1, and HHIP and immune cells, mainly focusing on Natural killer cells, CD8/CD4 T cells, and Immature B cells. It has been reported that activated natural killer cells are involved in the pathological process of IVDD (65). Further studies revealed that natural killer cells mainly have cytotoxic effects on NP cells (66). During IVDD, the protruding IVD tissue initiates an immune response, with the adaptive immune response being primarily manifested by the activation of T cell and B cell subsets (67). CD4 T cell subsets are involved in the regulation of inflammatory responses and offer potential targets for immunotherapy in IVDD (68). Upon activation, B cells produce antibodies, which are involved in the immune regulation and inflammatory response of IVDD (67). However, aberrant T-cell differentiation can result in the overexpression of inflammatory cytokines and the aberrant expression of B cells, both of which are critically linked to IVDD (67, 69).

Furthermore, we have identified potential therapeutic candidates for IVDD. DGIdb employs a data-mining approach to identify potential therapeutic targets or priority drug development based on specific genes (70). A total of 52 potential drugs for the treatment of IVDD were identified based on DGIdb. The majority of these drugs target the MET gene, followed by the CSF1 gene. Mesenchymal to epithelial transition factor (MET), also known as Cellular-mesenchymal to epithelial transition factor (c-Met) or Hepatocyte growth factor receptor (HGFR), is a member of the Protein tyrosine kinases family (71). c-MET is a specific receptor for hepatocyte growth factor (HGF). It has been reported that c-MET is expressed in NP cells, and activation of signaling by HGF binding to c-MET promotes cell proliferation and exerts anti-inflammatory effects (72). During IVDD, inflammatory stimuli enhance c-MET expression, while HGF treatment leads to decreased c-MET expression. Meanwhile, HGF treatment enhances hypoxia-inducible factor-1α (HIF-1α) expression to promote NP cell proliferation (73). In addition, activation of HGF/c-MET signaling inhibits the elevation of cyclooxygenase-2, MMP-3, and MMP-9 in NP cells after TNF-α stimulation (72). It has been demonstrated that apoptosis and ECM degradation play key roles in the progression of IVDD (74, 75). Thus, it is evident that HGF targeting c-MET has a protective effect on NP cells and can delay the process of IVDD by promoting cell proliferation and inhibiting the degradation of ECM. Colony-stimulating factor-1 (CSF1), also known as macrophage colony-stimulating factor (M-CSF), controls macrophage production, differentiation, and function (76, 77). Patients with IVDD are infiltrated with a large number of inflammatory cells, of which macrophages are the main inflammatory cells capable of infiltrating into the NP, and the number of macrophages is positively correlated with the grade of IVDD (66, 78). Macrophages that migrate to degenerating intervertebral discs (IVDs) differentiate into distinct cellular phenotypes (M1 or M2 type) in response to the local microenvironment. This differentiation process is regulated by a number of factors. It was demonstrated that p38 activation in NP cells could induce macrophage differentiation towards the M1 phenotype by modulating the local pro-inflammatory microenvironment. Conversely, blocking p38 activation in NP cells could inhibit M1 phenotypic differentiation and reduce CSF1 and IFN-γ secretion by NP cells, while simultaneously neutralizing CSF1 and IFN-γ-induced macrophage differentiation towards the M1 phenotype (79). In addition, CSF1 is also associated with microglial activation and proliferation. When NP is exposed to the dorsal root ganglia of the spinal cord, there is an upregulation of CSF1 expression in the dorsal root ganglia of the spinal cord and CSF1 receptor (CSF1R) in spinal cord microglia, which are activated at this time, and this behavior contributes to the pathogenesis of discogenic back pain through central sensitization (76). In summary, MET and CSF1 may be potential therapeutic targets for IVDD, but their specific mechanisms in IVDD development and progression require further experimental elucidation and assessment. Additionally, the drugs targeting these two genes based on theoretical prediction also need to be verified by more in-depth animal experiments and clinical trials after elucidating the mechanism of action of the two genes.

Recently, non-coding RNAs have been identified as pivotal regulators of gene expression (80). According to reports, non-coding RNAs can modulate the activation of autophagy in immune cells, thereby participating in the mediation of IVDD by directly or indirectly targeting autophagy-related genes and associated signaling pathways (81–83). Furthermore, lncRNA, miRNA, and mRNA can form ceRNA networks to regulate the occurrence and progression of IVDD (84, 85). We constructed potential ceRNA regulatory networks targeting these five model genes, providing new insights into the regulatory mechanisms and targeted therapies for IVDD. Within the constructed ceRNA network, multiple nodes have been reported to participate in the regulation of intervertebral disc degeneration (IVDD). LINC00969 is highly expressed in NP tissues and cells of IVDD patients, promoting IVD degeneration by sponging miR-335-3p and modulating NLRP3 inflammasome activity (86). In NP cells, overexpression of LINC00689 mediates autophagy via the miR-3127-5p/ATG7 axis, facilitating NP cell proliferation and suppressing apoptosis (87). NR2F1-AS1 is upregulated in IVD tissues of IVDD patients or NP cells treated with IL-1β or TNF-α, exacerbating IL-1β-induced extracellular matrix degradation and NP cell apoptosis, and regulates IVDD progression through miR-145-5p-mediated FOXO1 signaling pathway (88).

Although our results show accurate diagnostic efficacy of the model genes and have been validated in external datasets, there remain some limitations that require clarification. First, the sample size of gene expression profiles obtained from public databases is slightly insufficient, and individual differences between samples may affect the generalizability of the results. Furthermore, although we used cross-validation in model training, the relatively small sample size may still lead to overfitting of the model, thus affecting the robustness and generalizability of the results. Therefore, we will increase the sample size in future studies to avoid these problems and thus improve the accuracy of model prediction. Secondly, the lack of detailed clinical characteristics in the acquired data, such as age, degree of IVDD graded by Pfirrmann, duration of the disease, affected IVD level, and IVDD type (spinal stenosis, degenerative spondylolisthesis, intervertebral disc disease), which limits us to further reveal the potential association between model genes and certain traits. In addition, the targeted drugs and ceRNA regulatory networks derived from the model genes are at the analysis and hypothesis stage, requiring further validation through *in vitro* and *in vivo* experiments. Future studies will involve the design of prospective research to gather more comprehensive and multidimensional data for the verification of our findings. Meanwhile, various molecular biology techniques will be employed to conduct *in vitro* and *in vivo* experiments, aiming to fully understand the role of model genes and their potential regulatory mechanisms in IVDD.

## 5 Conclusions

In this research, four machine-learning methods were used to screen five model genes associated with lymphatic vessels, namely MET, HHIP, SPRY1, CSF1, and TOX. A diagnostic model with high predictive value was constructed based on these genes, effectively identifying patients with IVDD. Furthermore, potential therapeutic drugs for these model genes were predicted, and a lncRNA-miRNA-mRNA regulatory network was developed. Through comprehensive analysis, the potential association between lymphatic vessels and the occurrence and progression of IVDD was explored. Further research is urgently needed to validate and elucidate the regulatory mechanisms between them. Ultimately, our research provides novel insights into the pathogenesis of IVDD and contributes to the discovery of new therapeutic targets.

## Data Availability

The datasets presented in this study can be found in online repositories. The names of the repository/repositories and accession number(s) can be found in the article/**Supplementary Material**.
